# Size-Dependent Photophysical Behavior of Low Bandgap Semiconducting Polymer Particles

**DOI:** 10.3389/fchem.2019.00409

**Published:** 2019-06-11

**Authors:** Tersilla Virgili, Chiara Botta, Marta M. Mróz, Laurie Parrenin, Cyril Brochon, Eric Cloutet, Eleni Pavlopoulou, Georges Hadziioannou, Mark Geoghegan

**Affiliations:** ^1^IFN-CNR Dipartimento di Fisica, Politecnico di Milano, Milan, Italy; ^2^Laboratory Istituto per lo Studio delle Macromolecole, CNR-ISMAC, Milan, Italy; ^3^Laboratoire de Chimie des Polymères Organiques (LCPO) UMR 5629, CNRS-Université de Bordeaux-Bordeaux INP, Pessac, France; ^4^Department of Physics and Astronomy, University of Sheffield, Sheffield, United Kingdom

**Keywords:** transient absorption spectroscopy, PCDTBT, nanoparticles, semiconducting polymers, optoelectronics

## Abstract

The photophysics of water and propan-1-ol suspensions of poly [N-9”-heptadecanyl-2,7-carbazole-*alt*−5,5-(4,7-di-2-thienyl-2′,1′,3′- benzothiadiazole)] (PCDTBT) nanoparticles and mesoparticles has been studied by ultrafast spectroscopy. High molar mass polymer (HMM > 20 kg/mol) forms nanoparticles with around 50 nm diameter *via* mini-emulsion post-polymerization, while low molar mass (LMM < 5 kg/mol) polymer prepared by dispersion polymerization results in particles with a diameter of almost one order of magnitude larger (450 ± 50 nm). In this study, the presence of excited-states and charge separated species was identified through UV pump and visible/near-infrared probe femtosecond transient absorption spectroscopy. A different behavior for the HMM nanoparticles has been identified compared to the LMM mesoparticles. The nanoparticles exhibit typical features of an energetically disordered conjugated polymer with a broad density of states, allowing for delayed spectral relaxation of excited states, while the mesoparticles show a J-aggregate-like behavior where interchain interactions are less efficient. Stimulated emission in the red-near infrared region has been found in the mesoparticles which indicates that they present a more energetically ordered system.

## Introduction

Semiconducting polymer particles and nanoparticles with optical properties are of considerable importance for applications in a diverse range of technologies including light-emitting diodes (Wong, 2017), solar cells (Zhou et al., 2014; Jana et al., 2017), biosensing (Wu and Chiu, 2013; Chan and Wu, 2015; Xu et al., 2015), and cancer phototherapy (Li et al., 2018; Meng et al., 2018). Advantages of polymer particles include generally good optically stability, relatively routine preparation through emulsion polymerization or precipitation routes (Pecher and Mecking, 2010), and ready functionalization for different applications (Feng et al., 2013).

Some work has addressed the size dependence of the optical properties of semiconducting polymer nanoparticles but there is no conclusive narrative linking size to optical properties. In some experiments considering nanoparticles of different diameters, there was only limited evidence of size-dependent optical behavior (Lin et al., 2017; Peters et al., 2018). In experiments concerning polythiophenes (Kurokawa et al., 2004; Lee et al., 2010) and a polyfluorene copolymer (Pras et al., 2010) it was observed that smaller particles induce a blue shift in the emission spectra. By way of contrast however, decreasing nanoparticle size has led to a red shift in emission spectra of a poly(*p*-phenylene vinylene) (PPV) derivative (Sun et al., 2014), an observation that is not supported by experiments in which a different PPV derivative were allowed to aggregate in solution (Grey et al., 2006). Aggregated particles are therefore expected to behave differently to individual particles and one can therefore suppose that chain conformation and packing within the particles play an important role in their optoelectronic behavior. Indeed, this conclusion was reached by considering size-dependent brightness, which increased with increasing particle size less than would be expected by considering the number of fluorophores present in the volume (Sun et al., 2014). Certainly, experiments reporting the crystalline behavior of poly(3-hexylthiophene) nanoparticles showed that chain ordering controlled the optical behavior of these polymers (Labastide et al., 2011; Nagarjuna et al., 2012).

Poly[*N*-9”-heptadecanyl-2,7-carbazole-*alt*-5,5-(4′,7′-di-2-thienyl-2′,1′,3′-benzothiadiazole)] (PCDTBT) is a promising low bandgap polymer for bulk heterojunction solar cells (Blouin et al., 2007, 2008). It is an alternating copolymer comprising benzothiadiazole, thiophene, and carbazole units and is remarkable for its exceptional thermal stability (Blouin et al., 2008). Nanoparticles of PCDTBT blended with ([6,6]-phenyl-C_71_-butyric acid methyl ester) (PC_71_BM) have been demonstrated in bulk-heterojunction photovoltaic devices (D'Olieslaeger et al., 2017; Prunet et al., 2018). Different methods are available to create such particles, including the preparation of a miniemulsion comprising PC_71_BM, PCDTBT, and a surfactant in an appropriate solvent (D'Olieslaeger et al., 2017; Parrenin et al., 2017) or by first dissolving the components (PCDTBT and PC_61_BM or PC_71_BM) in a good solvent (tetrahydrofuran, THF) which is then dispersed in an excess of a non-solvent, water (Wang et al., 2016; Prunet et al., 2018). After the THF has been allowed to evaporate, a nanoparticle dispersion remains. It is however possible to prepare PCDTBT as particles of different sizes using different concentrations of the polymer in THF solution added to water (Parrenin et al., 2017). Particles of different optical sizes have been produced using a Suzuki cross-coupling dispersion polymerization, which were stabilized by a polymeric surfactant (Parrenin et al., 2015). PCDTBT nanoparticles can also be produced by the same routes as for the blends in the absence of the fullerene acceptor (Wang et al., 2016; Parrenin et al., 2017; Prunet et al., 2018).

Ultrafast transient absorption spectroscopy has previously been used to study the optical properties of PCDTBT in solution, in film form, and in fullerene blends. In thin films and in solution, relaxation from the π-π^*^ interband transition is seen to be similar and rapid (Banerji et al., 2010), with an initial relaxation process occurring on time scales less than the ~100 fs resolution of the experiment. Although a singlet exciton is formed within 1 ps, this is rather slow compared to the charge separation timescales occurring in blends with fullerenes (Tong et al., 2010). However, further transient absorption experiments were able to show that charge-transfer intermediate states do not play a significant role in the generation of photocurrent in the blends and that free charge carriers are generated directly and rapidly. Why Coulombically-bound excitons are bypassed in these systems is unresolved, but recent work has shown (using a modified transient absorption set-up) that there is some ordering at heterojunction interfaces, which may be responsible for the ability to create free charges after photoexcitation (Jakowetz et al., 2017).

Here, ultrafast spectroscopy was used to study the photophysics of water and propan-1-ol suspensions of different (PCDTBT) particle size in two forms: high molar mass (HMM) nanoparticles and larger mesoparticles of low molar mass (LMM). We have found that the HMM nanoparticles exhibit typical features of an energetically disordered conjugated polymer with a broad density of states, allowing for delayed spectral relaxation of excited states, while the LMM mesoparticles show a J-aggregate-like behavior where interchain interactions are less efficient. Moreover, stimulated emission in the red-near infrared region has been found in the LMM particles, which results from a more energetically ordered system.

## Materials and Methods

### Samples

**Figure 1:**
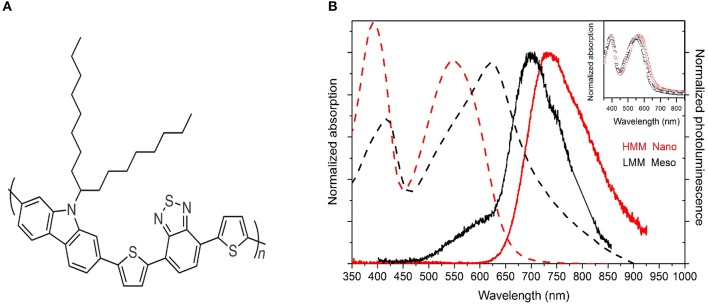
**(A)** Chemical structure of PCDTBT. **(B)** Normalized absorption (broken line) and photoluminescence spectra (solid line) for the HMM nanoparticle (red) and LMM mesoparticle (black) suspensions. The absorption spectra of spin coated films from the HMM (red open circles) and LMM (black open squares) pristine polymers are also shown in the inset. The pristine polymers are in film form after spin-coating from chloroform solution.

**Figure 2:**
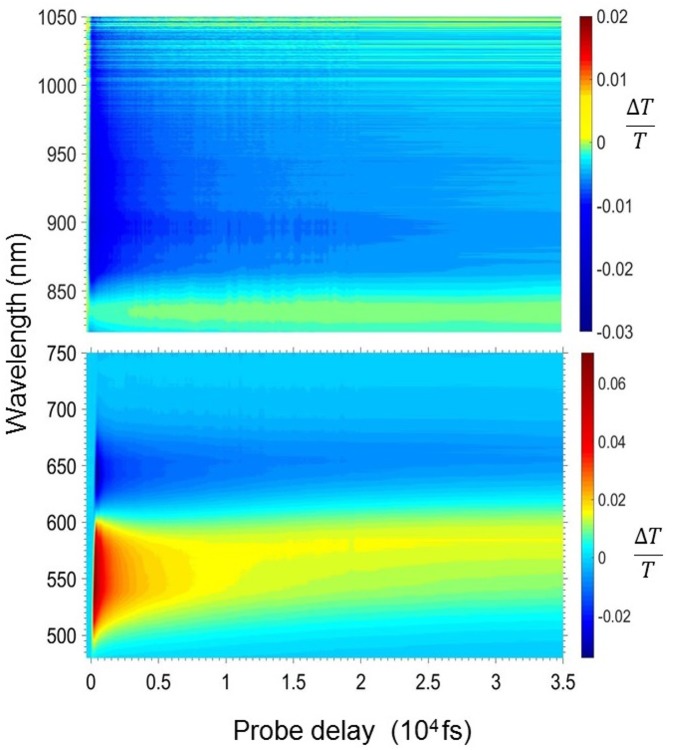
2-Dimensional Δ*T*/*T* map in the visible (bottom panel) and the near infrared (top panel) region for the HMM nanoparticle suspension.

Two routes to create PCDTBT particles (see Figure 1A for chemical structure) were used. High molar mass (HMM) nanoparticles were created as previously described (Parrenin et al., 2017). PCDTBT was first synthesized (Wakim et al., 2009) before being dissolved in chloroform. This was then added to an aqueous solution of sodium dodecyl sulfate (SDS) and sonicated to form a miniemulsion. Heating at 70°C allowed the chloroform to evaporate, leaving a nanoparticle dispersion in water. The PCDTBT was determined to have number-average molar mass, *M*_n_ = 20.2 kDa, and a dispersity of 2.2. The mean particle diameter was determined by transmission electron microscopy to be 50 nm (Figure 2S). Low molar mass (LMM) PCDTBT mesoparticles were created as previously described (Parrenin et al., 2015). PCDTBT was synthesized by Suzuki cross-coupling polymerization in a propanol solution with poly(vinyl pyrrolidone) (PVP) added as a surfactant. The quantity of PVP allowed the particle size to be adjusted in the range of 0.33–1.3 μm (Figure 1S), with an almost completely uniform distribution. The PCDTBT number-average molar mass was determined to be 4.5 kDa, with dispersity 2.1. Chloroform solutions (40 mg/mL) were prepared by dissolving the two different particles and then thin solid-state films of the two HMM and LMM polymers are prepared by spin coating.

### Steady State Absorption and Photoluminescence Spectra

Absorption and emission spectra were acquired using a Shimadzu UV-3600 spectrophotometer and a Horiba Scientific Fluoromax-4 spectrofluorometer, respectively. The excitation wavelength for the emission spectra was 390 nm.

### Ultrafast Spectroscopy

Time-resolved measurements were performed using a homebuilt femtosecond pump–probe setup (Virgili et al., 2013). A Ti:sapphire regenerative amplifier (Libra, Coherent) was used as a laser source, delivering 100 fs pulses at a central wavelength of 800 nm with 4 mJ pulse energy at a repetition rate of 1 kHz. For the excitation pulses, the second harmonic of the fundamental wavelength has been used (λ = 400 nm). In order to minimize bimolecular effects, the excitation density was kept to ~1.2 mJ cm^−2^. White light generated with a 2 mm-thick sapphire plate was used as a probe in the visible-near infrared range from 450 to 1,100 nm (the region between 750 and 820 nm cannot be detected due to the strong presence of the fundamental laser excitation). For a spectrally-resolved detection of the probe light, spectrographs and CCD arrays were used. The chirp in the white light pulse (the zero delay between the pump and the probe beams is different for different probe wavelengths) was taken into account during the analysis and evaluation of the two-dimensional (wavelength and time) Δ*T*(λ,τ)/*T* maps before extraction of the spectral and temporal data using homemade software. Overall, a temporal resolution of at least 150 fs was achieved for all excitation wavelengths.

## Results and Discussion

### Steady-State Spectra

PCDTBT is characterized by a relatively large donor-acceptor repeat unit. The absorption spectrum consists of several broad bands, which are ascribed to the lowest π-π^*^ interband transition, as is commonly observed in donor-acceptor copolymers of similar chemical structure (Westerling et al., 2007). The emission instead occurs with a large Stokes shift caused by the dissipation of energy in excess of equilibrium in photoexcited carriers during their relaxation, vibrational relaxation, localization of the excitation, exciton formation, exciton migration, and structural relaxation (Westerling et al., 2007; Banerji et al., 2010; Lin et al., 2017). The final state is a localized ground-state exciton.

Figure 1B shows the absorption and emission spectra of the two different particle suspensions. The absorption spectrum of the HMM nanoparticles (red broken line) is similar to that of previously reported PCDTBT films, exhibiting two broad (around 480 meV) transitions with peaks at 390 (more intense) and at 550 nm (less intense). In agreement with earlier work (Banerji et al., 2010; Etzold et al., 2011) the two bands were identified as the π-π^*^-transition of the first and second excited singlet states (S_1_ and S_2_). Excitation into the higher-energy band at 400 nm leads to a broad and unstructured emission between 620 and 920 nm (red solid line), peaking at 735 nm in the steady-state spectrum with a Stokes shift of around 185 nm corresponding to 570 meV.

**Figure 3:**
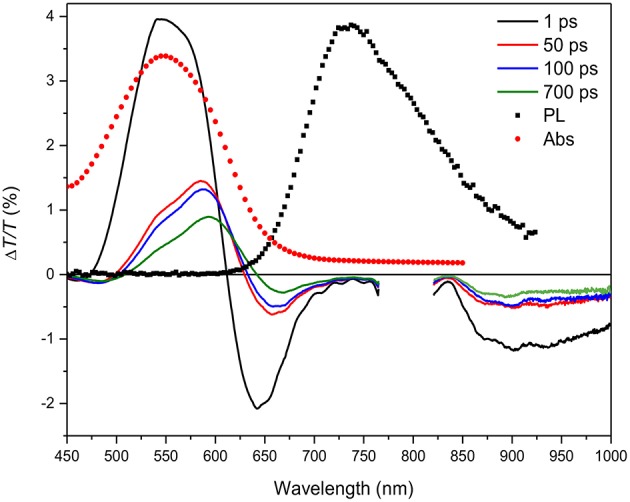
Δ*T*/*T* spectra at different probe delays (see legend in the figure) compared to the absorption (Abs, red solid circles) and photoluminescence (PL) emission (black solid squares) spectra for the HMM nanoparticle suspension.

**Figure 4:**
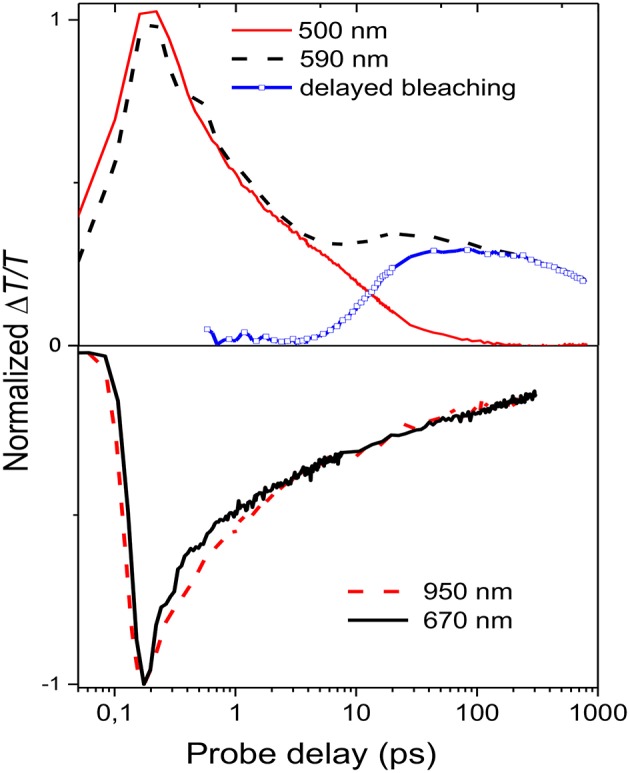
Normalized decay traces at different wavelengths (see legend in the figure) The blue line representing the delayed bleaching is calculated by subtracting the normalized decay at 500 nm from the one at 590 nm.

The absorption spectrum of the LMM suspension (black broken line) is quite different from those previously reported for PCDTBT (Banerji et al., 2010; Etzold et al., 2011). In contrast to what would be expected from considering the lower molar mass of the polymer (Shao and Vanden Bout, 2017), both transition peaks are red-shifted by around 150 meV, and also their relative intensity is changed, with the transition at 620 nm being more intense than that at 410 nm. The long wavelength absorption tail is likely to be due to the Rayleigh scattering of light from the particles (McQuarrie, 2000). The photoluminescence (PL) emission (black solid line in Figure 1B) surprisingly shows a Stokes shift of only 230 meV; in fact, the main peak of the emission is at 700 nm. The emission shows also a weak shoulder at around 580 nm, probably due to residual PVP surfactant (see Figure 3S) and/or dimers (Scarongella et al., 2013). The absence of energy transfer to the PCDTBT polymer despite the strong overlap between the absorption and emission of the two compounds reveals that those materials are in the suspension and not inside the nanoparticles so that their presence can be neglected in the following photophysical study. Moreover, μRaman measurements were performed on the particles and the presence of PVP was not detected (Figure 4S). The red-shift of the LMM PCDTBT mesoparticle absorption spectrum together with the strong reduction of the Stokes shift with respect to the HMM PCDTBT nanoparticle suspension indicate a different packing of the polymer chains.

**Figure 5:**
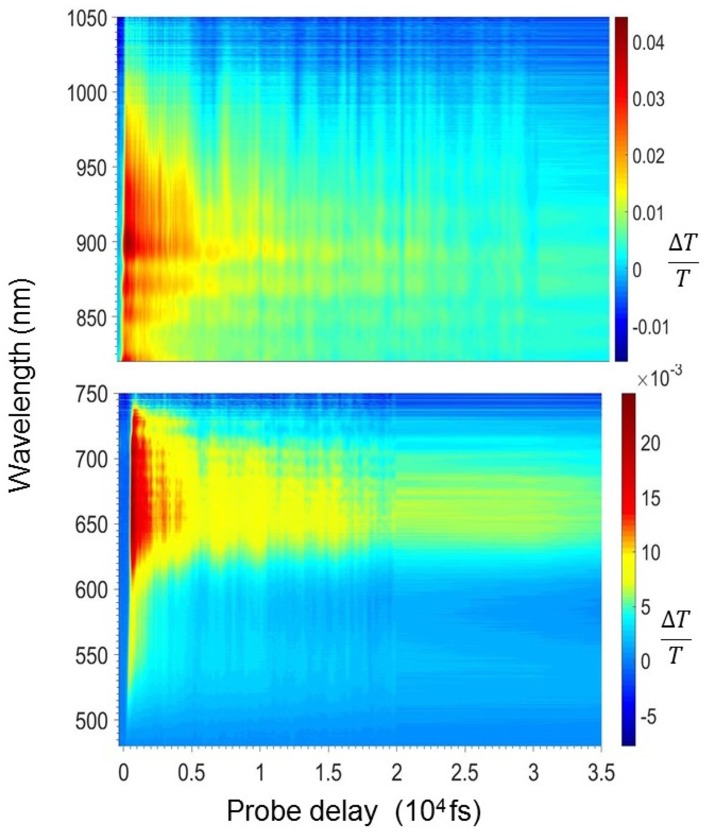
2-Dimensional Δ*T*/*T* map in the near infrared (bottom panel) and the visible (top panel) region for the LMM mesoparticle suspension.

To determine whether this behavior is due to the different molar mass of the polymers, HMM, and LMM particles were dissolved in chloroform and then spin coated to prepare uniform thin films. The absorption spectra of the two films are reported in the inset of Figure 1B. The spectra are very similar indicating that differences in particle optical properties can be attributed only to the different particle sizes rather than to their different molar mass or synthetic routes. Transient transmission measurements were also performed on those spin coated films (Figure 5S), which did not show significant differences in the photophysics of the two polymers. Any contribution due to molar mass is discussed below in the Discussion subsection.

The differences in the steady-state absorption-emission spectra of polymeric assemblies are commonly analyzed within an HJ-aggregate model (Spano, 2010; Baghgar et al., 2014; Ziffer et al., 2018). According to this model the different coupling types are spectroscopically identified by the intensity ratio between 0–0 and 0–1 vibrational transitions where the integer numbers refer to the vibrational quanta in the respective initial and final states. This ratio is larger (smaller) than unity for intrachain (interchain) coupling, i.e., J-like (H-like) behavior.

Here for the HMM suspension absorption spectrum, the π-π^*^-transition of the first singlet state S_1_ is a broad band where it is not easy to identify the 0–0 and the 0–1 vibrational transitions. However, it is clear that for the LMM suspension the main absorption peak is red-shifted with respect to the HMM suspension while the PL peak is blue-shifted as expected for J-aggregate-like behavior, which is generally associated with stronger intrachain than interchain coupling. From the optical properties it appears therefore that the mesoparticles obtained with the LMM polymer possess larger intrachain electronic coupling between monomer repeat units, probably due to a higher backbone planarity.

### Ultrafast Spectroscopy

#### Nanoparticles

Figures 2–4 show the photophysics of the HMM nanoparticle suspension. Figure 2 presents the 2D map of the transient transmission Δ*T*(λ,τ)/*T* in the visible (470–750 nm) and the near infrared spectrum (820–1,050 nm) in the first 35 ps after excitation. The measured signal is

$$\begin{array}{l} \frac{\Delta T\left(\lambda ,\tau \right)}{T}=\frac{T_{\text{pump}}-T_{\text{no pump}}}{T_{\text{no pump}}}, \end{array}$$

where *T*_pump_ and *T*_no pump_ are the probe transmission intensities after and before the pump excitation. A yellow-red signal represents a positive Δ*T*/*T* signal corresponding to a bleaching of the ground state or stimulated emission (SE) from excited states, while blue signal indicates the presence of a photoinduced absorption (PIA) band. The 2D map shows a positive band between 500 and 620 nm and two photoinduced absorption bands at around 650 and 900 nm.

The Δ*T*/*T* spectra, shown in Figure 3, display the temporal evolution of the three bands at four probe delays after the pump pulse. A comparison with the absorption and photoluminescence spectra allows discrimination between the signal due to ground-state bleaching and the excited-state stimulated emission. The positive signal with peak at 550 nm due to the overlap with the absorption spectrum (red filled circles) is assigned to the bleaching while the negative signals are attributed to polaron states as already reported (Banerji et al., 2010; Etzold et al., 2011).

Figure 4 shows the decay time traces at 500 and 590 nm corresponding, respectively, to the higher and lower energy exciton in the bleaching band and at 670 and 950 nm representing the peaks of the two absorption bands. It is noteworthy that the higher energy exciton (solid red line) presents one decay with a time constant of around 7 ps, while the one at 590 nm (broken black line) has two contributions: one fast decay similar to the one at 500 nm and a second contribution which grows at later time. To extrapolate only the second decay, the normalized decay at 500 nm was subtracted from that at 590 nm. The result, shown as the blue line in Figure 4, indicates that the lower energy exciton presents a component which is not instantaneous but due to energy transfer from higher energetic sites with a diffusion time of around 40 ps. The time traces at 670 and 950 nm are similar, which indicates that the two photoinduced absorption bands are related to the same population of charged states and that they are created instantaneously within the experimental time resolution (150 fs).

It has been previously shown that the long tail in the PCDTBT absorption spectrum indicates the presence of localized states near the band edge (Banerji et al., 2010). Those states can be seen as spectroscopic units of polymeric segments with different conjugation lengths; after photoexcitation the presence of those states can produce an energy transfer from higher to lower energetic states. Once formed the singlet excitons can hop to and between localized states and change their spatial position and the exciton hopping typically leads to a progressive red shift of the fluorescence spectrum, which slows in time as the number of nearby states with lower energy decreases (Banerji et al., 2010). In the nanoparticle suspension, the excitation energy transfer is from the higher energy units (polymer segments with a shorter conjugation length) to lower energy units (longer conjugation length) as represented by the decay traces at 500 and 590 nm (Figure 4).

#### Mesoparticles

**Figure 6:**
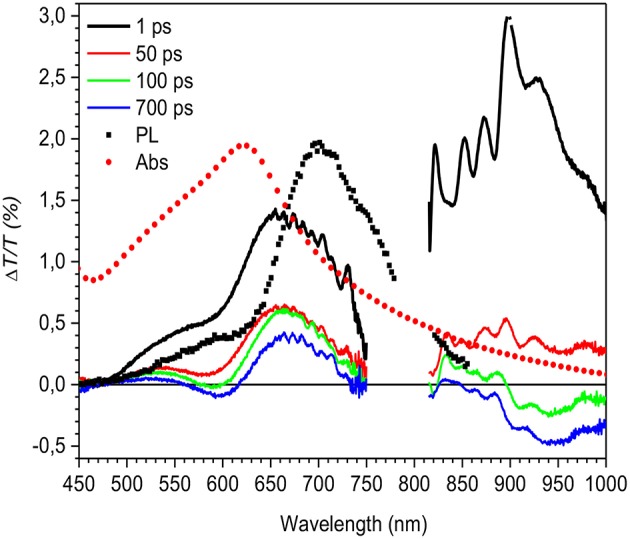
Δ*T*/*T* spectra at different probe delays (see legend in the figure) compared to the absorption (Abs, red solid circles) and photoluminescence (PL) emission (black solid squares) spectra for the LMM mesoparticle suspension.

**Figure 7:**
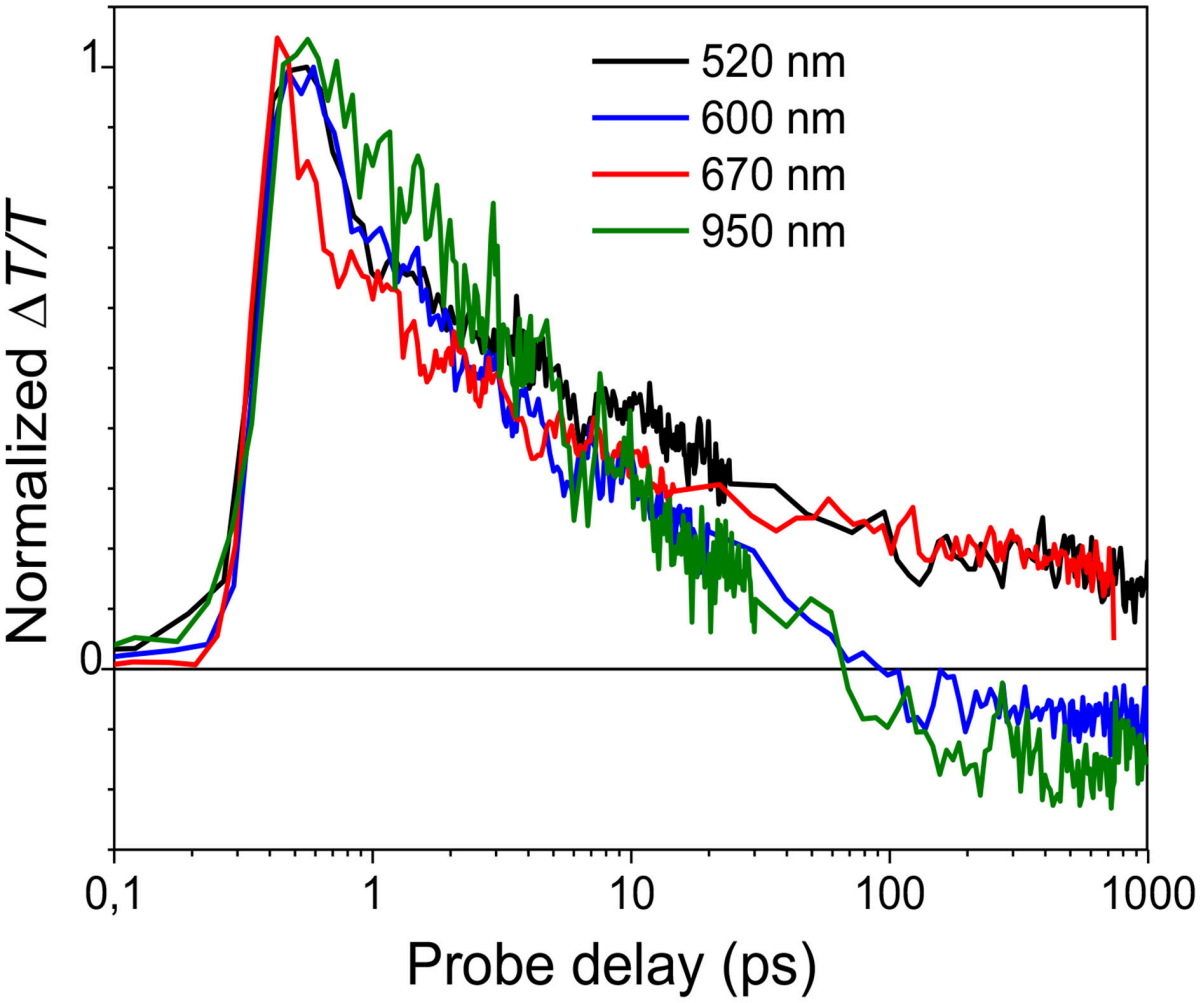
Normalized decay traces at different wavelengths (see legend in the figure) for the LMM mesoparticle suspension.

Figures 5–7 show the photophysics of the LMM mesoparticle suspension. Figure 5 presents the 2D map of the Δ*T*/*T* signal in the visible (470–750 nm) and the near infrared spectral region (820–1,050 nm) in the first 35 ps after excitation. Again, the yellow-red signal represents bleaching or stimulated emission, while the blue data are negative Δ*T*/*T* due to photoinduced absorption bands. The map is substantially, in the first 35 ps, a positive band ranging from 500 to 1,000 nm.

Figure 6 shows the transient transmission spectra at different probe delays. The spectra at 1 ps probe delay is positive in the entire spectral region, and by comparing the spectrum with the absorption and the photoluminescence data, it can be concluded that the signal is an overlap between the bleaching of the ground state and stimulated emission. Increasing the probe delay (green line in Figure 6) results in a new photoinduced absorption band that appears at ~600 and at 950 nm, and is attributed to charged states as previously reported (Banerji et al., 2010; Etzold et al., 2011; Provencher et al., 2014). Surprisingly the positive band extends until the near infrared region where a contribution from stimulated emission is evident. This can be concluded because the absorption signal is much less significant in this region. The overlap of the SE with the PIA band due to the photogenerated charges in this spectral region makes the PL efficiency very low and consequently undetectable in the PL measurements. As an aside, it is important to note from the transient transmission data that there is no indication of a signal coming from residual PVP surfactant present in the solution, as occurred in the photoluminescence spectrum, which indicates that its presence is negligible.

To confirm the origin of the positive transient signal, the dynamics at 520, 600, 670, and 950 nm are compared in Figure 7. The decays are similar in first 10 ps indicating that the decay of the singlet exciton states is the origin of the photoinduced signal. After 10 ps, Δ*T*/*T* at 600 and 950 nm becomes negative; the positive signal at these wavelengths overlaps with a negative photoinduced absorption band that is attributed to charged-state formation as previously reported (Provencher et al., 2014).

### Discussion

The transient transmission spectra for the different particle suspensions, as for the steady state absorption and emission spectra, are completely different. While the small polymer particles present a photophysics similar to a disordered polymer film, the large particles behave in a completely different way. There are three important differences: Firstly, in the LMM mesoparticle suspension there is *no indication* of the typical PCDTBT exciton energy transfer from the higher energetic states to the lowest state while in the nanoparticle suspension this effect is evident (see blue line in Figure 4). Secondly, the signal from the charged states (blue line in Figure 7) is weak and is not instantaneous in the LMM suspension while the charges are instantaneously generated in the HMM suspension (see black solid line in Figure 4). Finally, stimulated emission occurs in the near infrared region only for the LMM suspension. All of these properties indicate a less efficient interchain interaction strengthening the idea that the LMM particle suspension has a more J-aggregate-like behavior that is not observed in the HMM-nanoparticle suspension.

It has been demonstrated in a range of different polymer systems that the strength of interchain vs. intrachain interactions, resulting in modulation between predominately H- vs. J-aggregate-like behavior, can be tuned systematically by parameters such as solvent casting and annealing (Khan et al., 2004; Eder et al., 2017), pressure (Niles et al., 2012), regioregularity (Clark et al., 2007), and molar mass (Paquin et al., 2013). From the perspective of classical polymer science, it is established that polymers of low molar mass form unconnected, extended-chain crystals. Due to the non-entangled nature of these relatively short-chain macromolecules, this leads to a polycrystalline, one-phase morphology. In contrast, with materials of molar mass larger than the entanglement molar mass, typically two-phase morphologies are obtained, which comprise crystalline moieties embedded in largely amorphous regions, whereby individual macromolecules bridge multiple domains of order (Wunderlich, 1976; Paquin et al., 2013). For poly(3-hexylthiophene) it was found that the macromolecules in aggregated regions of high molar mass polymer adopt a more planar conformation compared to low molar mass materials. This results in the observed increase in intrachain exciton coherence. In contrast, shorter chains seem to lead to more disordered architectures (Paquin et al., 2013). In contrast to those previous results, here the low molar mass particle suspension presents a J-aggregate-like behavior (i.e., more intrachain interaction) but not the high molar mass suspension.

These results indicate that the means of particle preparation affects not only the particle size but also the packing of the polymer chains inside the particles, which dramatically changes their spectroscopic properties. Given that the optical properties of the thin films have been shown to be similar (Figure 1B and Figure 5S), it is unlikely that the molar mass plays a significant role. It is known that polymer molar mass does affect crystallization (Cheng et al., 1992), and were this to be a factor in the preparation process, then low molar mass polymers may be less well-stacked or less planar than their larger counterparts due, for example, to free chain ends, which would increase the likelihood of localized excitons and greater energetic order. It is nevertheless difficult to envisage how particle size alone can control whether or not excitons are localized, and so the possibility that the method of preparing the PCDTBT particles plays a role cannot be discounted.

## Conclusion

The photophysics of poly[N-9′-heptadecanyl-2,7-carbazole- *alt*−5,5-(4,7-di-2-thienyl-2′,1′,3′- benzothiadiazole] (PCDTBT) nanoparticle and mesoparticle suspensions has been investigated. The nanoparticles present a similar behavior of the pristine polymer, with a large Stokes shift and the presence of many localized states near the band edge. The efficient energy transfer from the higher energetic state to the lowest one happens in a time constant of around 7 ps. In these nanoparticles no stimulated emission is detected due to the presence of a photoinduced absorption band related to charged states which overlap the stimulated emission spectral region. The mesoparticles have a completely different behavior. The peak of the absorption spectrum is red-shifted and narrower than that of the nanoparticle suspension, and the Stokes shift is reduced as well as the efficiency of charges formation indicating a more evident J-aggregate-like behavior (fewer interchain interactions) compared to the nanoparticle suspension. Moreover, stimulated emission is detected in the near infrared region for the mesoparticle suspension. It is concluded that the method of particle preparation is crucial not only to determine the particle size but also to change the packing state of the polymer backbones that strongly affects the photophysical properties of the PCDTBT polymer.

## Author Contributions

The project was devised by TV and MG and supervised by TV, MG, and EP. Data contained in the paper were obtained and analysed by TV, MM, and ChB in Milan. Micro-Raman spectroscopy and preparatory and comparative absorption and photoluminescence data were obtained and analysed in Bordeaux by LP, EP, CyB, and EC. All authors contributed to the scientific discussion of the results. The manuscript was written by TV and MG, although all authors contributed and approved the final version.

## Conflict of Interest Statement

The authors declare that the research was conducted in the absence of any commercial or financial relationships that could be construed as a potential conflict of interest.
