# Supplementary material for: Size-Dependent Photophysical Behavior of Low Bandgap Semiconducting Polymer Particles
Source: Front Chem. 2019 Jun 11;7:409. doi: 10.3389/fchem.2019.00409 (PMC6584897; doi:10.3389/fchem.2019.00409)
Supplement: Supplementary file 1 [file Data_Sheet_1.pdf]

Supplementary Information

Size-Dependent Photophysical Behaviour of Low Bandgap Semiconducting Polymer Particles

Tersilla Virgili[1*], Chiara Botta[2], Marta M. Mroz[1], Laurie Parrenin[3], Cyril Brochon[3], Eric Cloutet[3], Eleni Pavlopoulou[3], Georges Hadziioannou[3], and Mark Geoghegan[4]

[1]IFN-CNR Dipartimento di Fisica, Politecnico di Milano, Milano 20132, Italy

[2]Laboratory Istituto per lo Studio delle Macromolecole, CNR-ISMAC, via A. Corti 12, 20133 Milano, Italy

[3] Laboratoire de Chimie des Polymères Organiques (LCPO) UMR 5629, CNRS-Université de Bordeaux-Bordeaux INP, B8 Allée Geoffroy Saint Hilaire, F-33615 Pessac CEDEX, France

[4]Department of Physics and Astronomy, University of Sheffield, Hicks Building, Sheffield S3 7RH, UK

TEM measurements

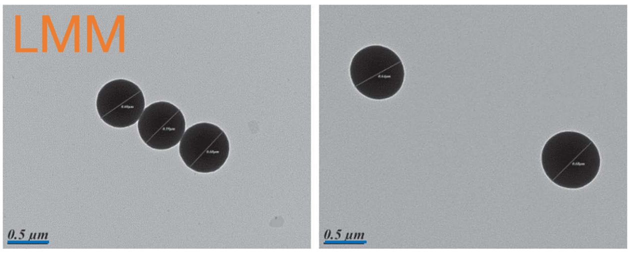

Figure 1S. Transmission electron microscopy (TEM) images for the LMM mesoparticles are reproduced with permission from (Parrenin et al., 2015). Copyright (2015) John Wiley & Sons.

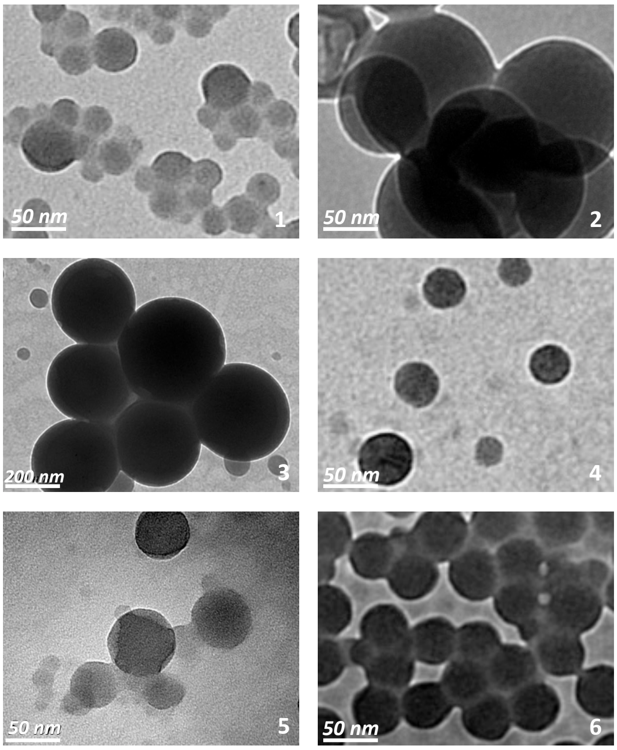

Figure 2S. TEM images for the HMM nanoparticles are reproduced with permission from (Parrenin et al., 2017). Copyright (2017) American Chemical Society.

Emission from the PVP surfactant

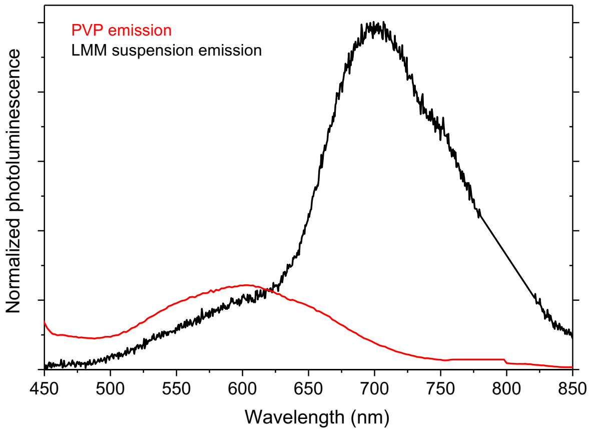

Figure 3S. Comparison between the PL emission of the LMM suspension and the PVP surfactant after excitation at 390 nm.

μRaman measurements on the LMM mesoparticles

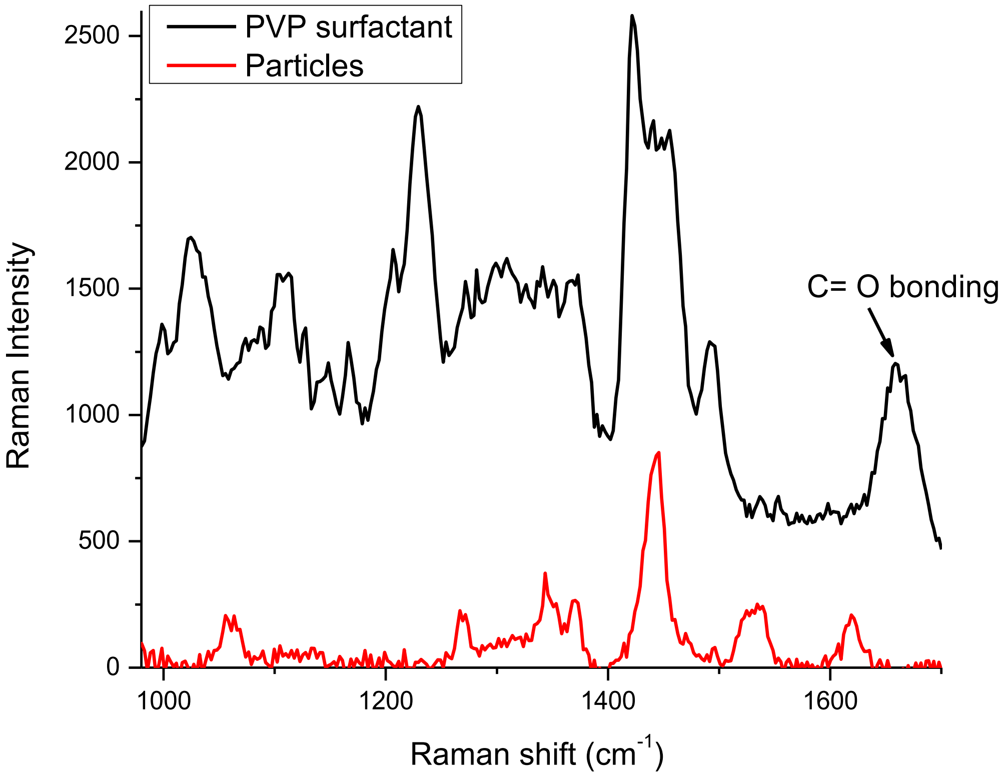

Figure 4S. μRaman spectroscopy measurements were performed on the LMM mesoparticles to check the presence of the PVP surfactant. The spectrum (red line) revealed no PVP presence (black line). The spectrum from the particles resembles that of an earlier one for PCDTBT (Provencher et al., 2014). These experiments were recorded in backscattering mode on a modified HR800 (HORIBA Jobin Yvon) confocal micro-Raman spectrometer. A continuous wave laser operating at 785 nm was used with typical resolution of 2.5 cm^{-1}.

Transient Transmission measurements on the LMM and HMM polymer spin coated films

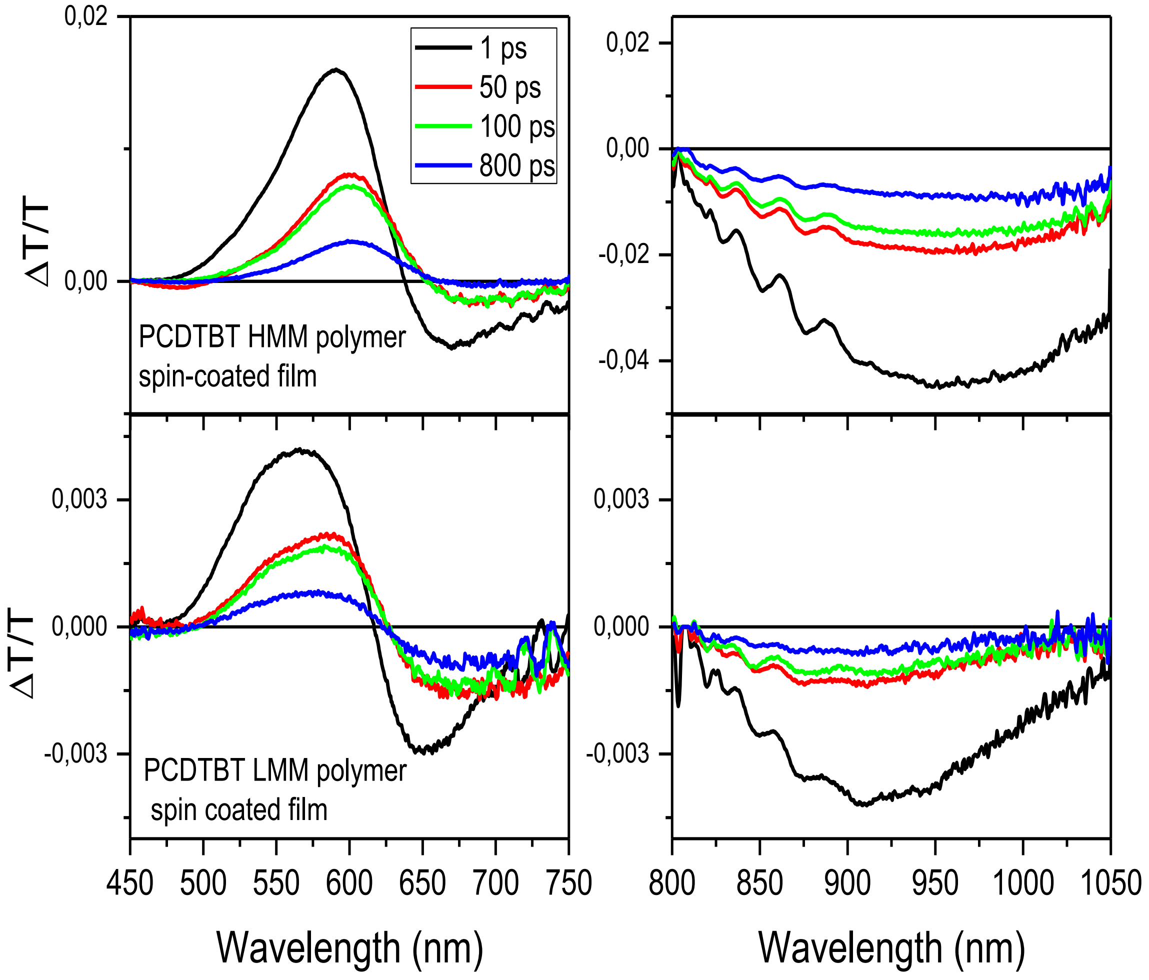

Figure 5S. Pump-probe spectra at different probe delays. In the top/bottom panels the spectra in the visible and near infrared region are shown for the HMM/LMM polymer spin coated films.

References

Parrenin, L., Brochon, C., Hadziioannou, G., and Cloutet, E. (2015). Low bandgap semiconducting copolymer nanoparticles by Suzuki cross-coupling polymerization in alcoholic dispersed media. *Macromol. Rapid Commun.* 36**,** 1816-1821.

Parrenin, L., Laurans, G., Pavlopoulou, E., Fleury, G., Pecastaings, G., Brochon, C., Vignau, L., Hadziioannou, G., and Cloutet, E. (2017). Photoactive donor-acceptor composite nanoparticles dispersed in water. *Langmuir* 33**,** 1507-1515.

Provencher, F., Bérubé, N., Parker, A.W., Greetham, G.M., Towrie, M., Hellmann, C., Côté, M., Stingelin, N., Silva, C., and Hayes, S.C. (2014). Direct observation of ultrafast long-range charge separation at polymer-fullerene heterojunctions. *Nature Commun.* 5**,** 4288.
